# Development of Digital Twins to Optimize Trauma Surgery and Postoperative Management. A Case Study Focusing on Tibial Plateau Fracture

**DOI:** 10.3389/fbioe.2021.722275

**Published:** 2021-10-07

**Authors:** Kévin Aubert, Arnaud Germaneau, Michel Rochette, Wenfeng Ye, Mathieu Severyns, Maxime Billot, Philippe Rigoard, Tanguy Vendeuvre

**Affiliations:** ^1^ Institut Pprime UPR 3346, CNRS – Université de Poitiers – ISAE-ENSMA, Poitiers, France; ^2^ Ansys France, Villeurbanne, France; ^3^ Department of Orthopedic and Trauma Surgery at the University Hospital Center of Fort-de-France, Fort-de-France, France; ^4^ PRISMATICS Lab (Predictive Research in Spine/Neuromodulation Management and Thoracic Innovation/Cardiac Surgery), Poitiers University Hospital, Poitiers, France; ^5^ Department of Spine Surgery and Neuromodulation, Poitiers University Hospital, Poitiers, France; ^6^ Department of Orthopedic Surgery and Traumatology, Poitiers University Hospital, Poitiers, France

**Keywords:** digital twin, finite element analysis, tibial fracture, personalized medicine, minimally invasive surgery

## Abstract

**Background and context:** Surgical procedures are evolving toward less invasive and more tailored approaches to consider the specific pathology, morphology, and life habits of a patient. However, these new surgical methods require thorough preoperative planning and an advanced understanding of biomechanical behaviors. In this sense, patient-specific modeling is developing in the form of digital twins to help personalized clinical decision-making.

**Purpose:** This study presents a patient-specific finite element model approach, focusing on tibial plateau fractures, to enhance biomechanical knowledge to optimize surgical trauma procedures and improve decision-making in postoperative management.

**Study design:** This is a level 5 study.

**Methods:** We used a postoperative 3D X-ray image of a patient who suffered from depression and separation of the lateral tibial plateau. The surgeon stabilized the fracture with polymethyl methacrylate cement injection and bi-cortical screw osteosynthesis. A digital twin of the patient’s fracture was created by segmentation. From the digital twin, four stabilization methods were modeled including two screw lengths, whether or not, to inject PMMA cement. The four stabilization methods were associated with three bone healing conditions resulting in twelve scenarios. Mechanical strength, stress distribution, interfragmentary strains, and fragment kinematics were assessed by applying the maximum load during gait. Repeated fracture risks were evaluated regarding to the volume of bone with stress above the local yield strength and regarding to the interfragmentary strains.

**Results:** Stress distribution analysis highlighted the mechanical contribution of cement injection and the favorable mechanical response of uni-cortical screw compared to bi-cortical screw. Evaluation of repeated fracture risks for this clinical case showed fracture instability for two of the twelve simulated scenarios.

**Conclusion:** This study presents a patient-specific finite element modeling workflow to assess the biomechanical behaviors associated with different stabilization methods of tibial plateau fractures. Strength and interfragmentary strains were evaluated to quantify the mechanical effects of surgical procedures. We evaluate repeated fracture risks and provide data for postoperative management.

## Introduction

For several years, orthopedic surgery has been moving toward less invasive approaches that consider the specific pathology, morphology, and life habits of patients. Tibial plateau fractures represent 1.03% of all bone fractures in the general population (Elsoe et al., 2015) and 8% in the elderly population (Rozell et al., 2016). Recently, a new minimally invasive surgery (MIS) named tuberoplasty has been developed to treat the two most common types of tibial plateau fractures: Type II and Type III in the Schatzker classification (Vendeuvre et al., 2013, 2019; Kfuri and Schatzker, 2018) also known as Type 41-B3.1 and Type 41-B2.1 in the OTA/AO classification (Meinberg et al., 2018).

The most frequent tibial plateau fracture is Schatzker Type II. It corresponds to a compression fracture resulting in the separation and the depression of the lateral condyle of the tibia (Kfuri and Schatzker, 2018). To treat this type of fracture, tuberoplasty consists in two phases, reduction and stabilization. Reduction consists in using an expanding balloon under depressed fragments to progressively raise them until the reconstruction of tibial plateau surface. Stabilization consists in consolidation of the reconstructed tibia using PMMA (poly methyl methacrylate), screws, and/or plate. The use of PMMA cement is motivated by its mechanical strength and its contribution to the stabilization (No et al., 2014). While the stabilization phase of tuberoplasty has experimentally shown its biomechanical interest in terms of mechanical strength (Vendeuvre et al., 2018), the stress distribution and fragment displacement associated with different variants of the stabilization method remain complex to quantify experimentally. To bridge this gap, finite element (FE) models was recently introduced to improve knowledge of mechanical behavior after proximal tibial surgery (Huang et al., 2015; Carrera et al., 2016, 2018; Belaid et al., 2018; Dehoust et al., 2020; Ingrassia et al., 2020).

In 2015, Huang et al. observed the stress distribution and stability of fixations, using finite element simulations, for the treatment of Schatzker IV tibial plateau fractures (medial condyle separation). By comparing screw only versus screw and plate stabilization, they observed an influence on the stress distribution in the bone and a higher stability for the plate and screw solution. In 2016, Carrera et al. compared stabilizations of tibial plateau fracture type Schatzker I (lateral condyle separation) with two cannulated screws versus the locking plate. They simulated full weight-bearing at time zero. Full weight-bearing corresponds to the first time the patient can walk again. While they found no clinical relevance on interfragmentary motion, they reported higher mechanical stability with the locking screw plate. The authors concluded that full or at least partial weight-bearing should be allowed with the locking plate at time zero. In their subsequent research, Carrera et al. (2018) showed that the integrity of the fibula and the proximal tibiofibular joint combined with a locking plate system may allow early weight-bearing for Schatzker type I tibial plateau fracture. Belaid et al. (2017) proposed to compare the stress distribution in the bone between a healthy tibia and a stabilized tibial plateau. The plateau was stabilized after a Schatzker II tibial plateau fracture (separation and depression of the lateral plateau). They observed a similar stress distribution on the plateau but a decrease in stress along the plate due to the support provided by the plate. In addition, they observed an increase in stress in the fracture zone. To continue this study, Belaid et al. (2018) evaluated the usefulness of cement injection for the stabilization of fracture type Schatzker II by modeling different clinical stage. They observed that cement injection ensures a stress distribution similar to a healthy tibia when the plate and screws are removed and the bone remodeled around the cement. In 2020, Dehoust et al. modeled and evaluated the stabilization of bi-condylar fractures (Type Schatzker V). Four stabilization methods were simulated, including the use of screws and/or plates. They were able to identify that additional plate provides better stability. Ingrassia et al. (2020) evaluated the influence of the position and orientation of the screws used for plate stabilization of Schatzker I fracture (lateral condyle separation). They observed the reaction forces and contact pressures between the screws and the bone. Although the most favorable orientation was determined for the case studied, no general conclusion was postulated.

According to our knowledge, no study has been published about the stabilization method influence on the stress distribution and the fracture stability at different stages of bone healing after tibial plateau fractures.

While for older patients it is desirable to provide stabilization allowing early full weight-bearing, for younger patients, priority can be given to reducing invasiveness. Nowadays, clinicians do not possess information enabling them to determine the surgical procedure to provide optimal stabilization according to patient characteristics and life habits. Our question is as follows: Can quantified biomechanical data from a patient-specific FE model improve surgical procedures and assist in clinical decisions making about postoperative treatment of tibial plateau fractures?

This study presents a method to build personalized FE models of bone trauma, the objective being to quantify the biomechanical interest of stabilization variants at different stages of bone healing. A digital twin approach based on a Schatzker type II tibial plateau fracture is proposed with consideration of patient-specific fragment geometries, material properties, and morphological loading. The digital twins of the patient’s fracture were used to evaluate the biomechanical interests of four stabilization methods in three bone healing conditions.

## Materials and Methods

Patient-specific finite element (FE) modeling requires four inputs that should all be subject-specific: the geometry of studied structures, the material properties of each structure, the boundary conditions, and loading (Scott et al., 2020).

### Geometry of Studied Structures

An *in situ* postoperative three-dimensional (3D) image of a tibial plateau fracture type Schatzker II was obtained from a 40-year-old patient (male, 80 kg and 1.82 m), in accordance with the ethics approbation (CHU86-RECH-R2020-03-02). The fracture reduction was made by MIS by balloon inflation according to the tuberoplasty technique (Vendeuvre et al., 2013). Surgeon performed a synthesis in accordance with the AO recommendations (Buckley et al., 2017): a standalone bi-cortical screw of 6.5 mm of diameter (Asnis 125 III cannulated screw, Stryker, Kalamazoo, Michigan, United States) was positioned in the metaphysis perpendicularly to the separation and under the depression fragment in order to ensure metaphysis compression. Stabilization was completed with a PMMA filling (Kyphon ^®^ Xpede™, Medtronic Inc., Dublin, Ireland). The 3D image was acquired in the operating room with a 3D X-ray CT-scan (O-ARM^®^ IMAGING SYSTEM, Medtronic) a few minutes after the surgery. The voxel size was 415 × 415 × 833 μm^3^ and the reconstructed matrix was 512 × 512 × 192 voxels. Bone fragments geometries and PMMA cement were segmented from the 3D image with 3D Slicer software (V4.10.1) (Kikinis et al., 2014) with a semi-automatic method based on a region-growing algorithm (Mehnert and Jackway, 1997).

**FIGURE 1:**
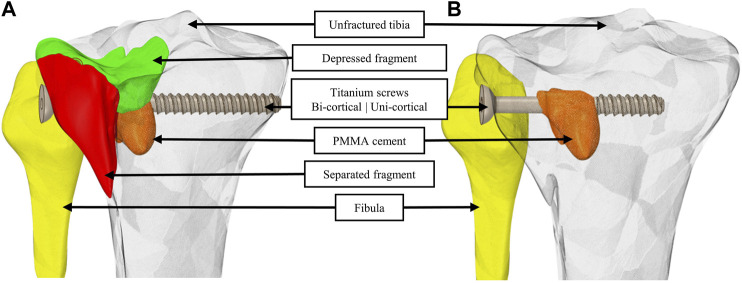
Geometric models with identification of the separated and the depressed fragments **(A)** and by considering bone fragments merged into a single geometry and interfragmentary gaps filled **(B)**. For a visual understanding, a bi-cortical screw is integrated to the model in **(A)** and a uni-cortical screw is integrated in **(B)**. PMMA cement is integrated in both **(A)** and (**B)**.

Two segmentations were produced from the image segmentation step; both were composed of the specific geometry of the tibia and the fibula. One segmentation has identification of the fractured bone (Figure 1A), while the other considered the tibial plateau as a single geometry to model an advanced state of bone remodeling with fragments fused and interfragmentary gaps filled (Figure 1B).

On each of the two segmentations, four stabilization variants were modeled (Table 1). The first variant was the one used by the surgeon for the clinical case. Additionally, we modeled three more stabilization variants presented Table 1. On the 3D image, we identified the axis of the bi-cortical screw used by the surgeon to import screws in models. Instead of segmenting the screws, we imported it to ensure better fidelity of the screw thread. This step results in eight geometrical models including two considerations of the bone fragments interface and four stabilization variants.

**TABLE 1:** Description of the 12 simulated scenarios including stabilization variant characteristics and bone healing conditions.

Stabilization variant					Geometrical models	Scenarios number - Bone fragment interactions - [Time after the surgery]			Observations
Screw	Diameter (mm)	Length (mm)	Thread length (mm)	Cement injection					
Bi-cortical Asnis III cannulated screw, Stryker	6.5	70	40	Yes	I	1	Mobile	[Before 3 weeks]	Stress fields displacement fields strain fields inter-fragmentary strains
						2	Bonded	[From 6 to 12 weeks]	
					II	3	Fused	[1 year ]	
				No	III	4	Mobile	[Before 3 weeks]	
						5	Bonded	[From 6 to 12 weeks]	
					IV	6	Fused	[1 year ]		
Uni-cortical Asnis III cannulated screw, Stryker	6.5	50	20	Yes	V	7	Mobile	[Before 3 weeks]	
						8	Bonded	[From 6 to 12 weeks]	
					VI	9	Fused	[1 year ]	
				No	VII	10	Mobile	[Before 3 weeks]	
						11	Bonded	[From 6 to 12 weeks]
					VIII	12	Fused	[1 year ]

### Material Properties of Each Structure

We used Simpleware™ software (version 2019.09; Synopsys, Mountain View, California, United States) to mesh geometric models and to allocate material properties. A convergence study was conducted to determine the optimal mesh parameters. The resulting meshes were composed of quadratic tetrahedral elements with a mean size of 0.64 mm. Meshes followed the requirements listed by Burkhart et al. (2013) in terms of aspect ratio, angle idealization, and Jacobian element for quadratic tetrahedral elements.

**FIGURE 2:**
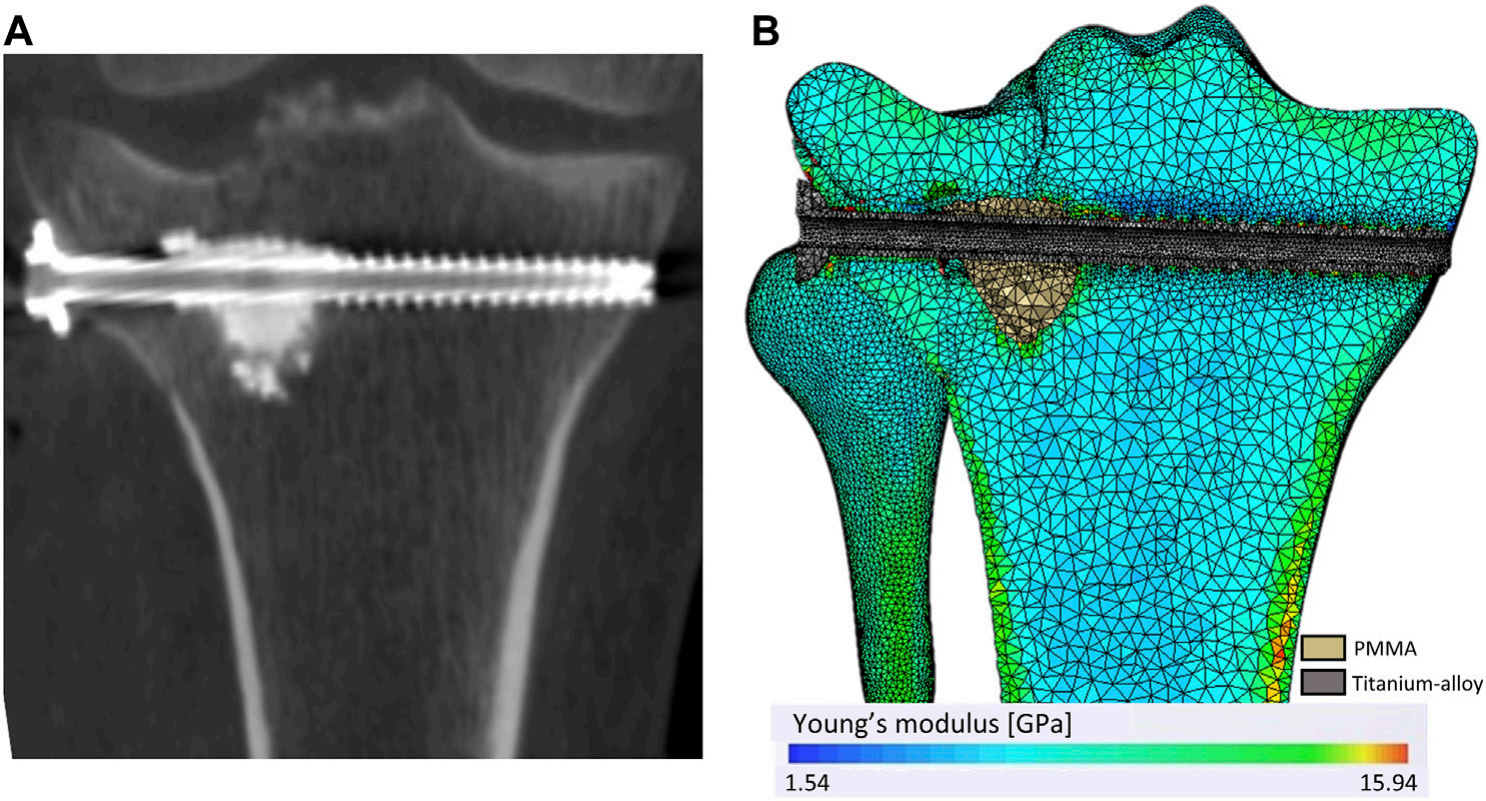
Mesh and heterogeneous distribution of bone material properties. **(A)** A coronal plan of the 3D X-ray image. **(B)** The mesh and the material properties distribution according to the bone density observe through the 3D X-ray image.

Local bone mineral densities (BMDs) observed on the 3D X-ray image were calibrated with a density phantom [CIRS-Model-062M]. The same acquisition parameters were used for the phantom and the patient. Once the mesh was superimposed on the 3D X-ray image, bone material properties were assigned on each mesh element depending on surrounding bone density (Figure 2). Table 2 presents the relationship between BMD and material properties for the cortical bone and the trabecular bone. For all materials, the Young modulus, Poisson’s ratio, and yield strength were defined (Snyder and Schneider, 1991; Rho et al., 1995; Lee, 2005; Cook et al., 2010; Havaldar et al., 2014; Nazemi et al., 2015; Belaid et al., 2018). The density limit to determinate the trabecular bone from the cortical bone was set at 1.68 g/cm^3^ to correspond to the intersection between the functions defining the Young modulus of the trabecular bone and the cortical bone.

**TABLE 2:** Material properties.

	Young modulus [MPa]	Poisson’s ratio	Yield strength [MPa]	References
Cortical bone	3,890×ρ_BMD_^2.39^	0.3	141.6	Snyder and Schneider (1991)
				Havaldar et al. (2014)
Trabecular bone	6,570×ρ_BMD_^1.37^	0.3	$\log\left(\sigma_{Trabecular\;Bone\;YS}\right)=1.38+1.91\times \log\left(\rho_{BMD}\right)\;$	Rho et al. (1995)
				Nazemi et al. (2015)
				Cook et al. (2010)
Titanium alloy screw	110,000	0.3	930	Belaid et al. (2018)
PMMA cement	2,163	0.375	93	Belaid et al. (2018)
				Lee (2005)

In scenarios using uni-cortical screw, the density of the bone replacing the end of the bi-cortical screw was set as 0.94 g/cm^3^. This value corresponds to the most frequent density observes in the tibial plateau of the clinical case.

### Boundary Conditions and Loading

Ansys Mechanical software (version 2020R2, Ansys, Canonsburg, Pennsylvania, United States) was used to define the boundary conditions and to perform simulations. In each scenario, the maximum load during gait was simulated. It was defined as 2.59 times the patient’s body weight distributed between the lateral and the medial plateau (37–63%) (Zhao et al., 2007). The surfaces on which the forces were applied have been located regarding to the contact area with the femur of the patient and sized in accordance with works from the literature (Poh et al., 2012). The resulting surfaces corresponded to 403 and 374 mm^2^ in the lateral and medial condyle, respectively. Fix supports were applied on nodes of the distal extremities of the tibia and fibula. The interaction between the fibula head and tibia was considered bonded.

Twelve scenarios of full weight-bearing were simulated combining four stabilization variants at three different bone healing conditions (Table 1). Mobile bone fragments, with a frictionless contact assumption, modeled early full weight-bearing practices when fracture healing is in an early stage, before the hard callus formation, which occurs 3 weeks after the surgery (Hofman et al., 2015). Bonded bone fragments, with a bonded contact assumption, modeled intermediary stage of fracture healing, during callus formation, which ends around six to twelve weeks after surgery. For scenarios with mobile bone fragments and bonded bone fragments, the geometries of the bone structures were identical, only the contact assumption is different (Figure 1A). For full bone remodeling, a year and more after the surgery, the fragments were considered as fused into a single geometry and interfragmentary gaps filled (Hofman et al., 2015) (Figure 1B). In all scenarios, the contacts that include cement or screw were considered bonded.

For mobile bone fragments and bonded bone fragments scenarios, the initial fragments positions corresponded to their postoperative position. Postoperative gap sizes were 1.78 mm for the depressed fragment and 3.5 mm for the separated fragment.

### Result Analysis

Mechanical strength, stress distribution ( von Mises), and the volume of bone with stress above the local yield strength were computed. Moreover, we evaluate the reduction loss, total displacements, and interfragmentary strains. Reduction loss was expressed as the displacement in the distal–proximal direction for the depressed fragment and as separate fragment opening in the medial–lateral direction. The interfragmentary strains were defined as the inter-fragmentary movement divided by the initial fracture gap size (Lacroix and Prendergast, 2002).

## Results

**FIGURE 3:**
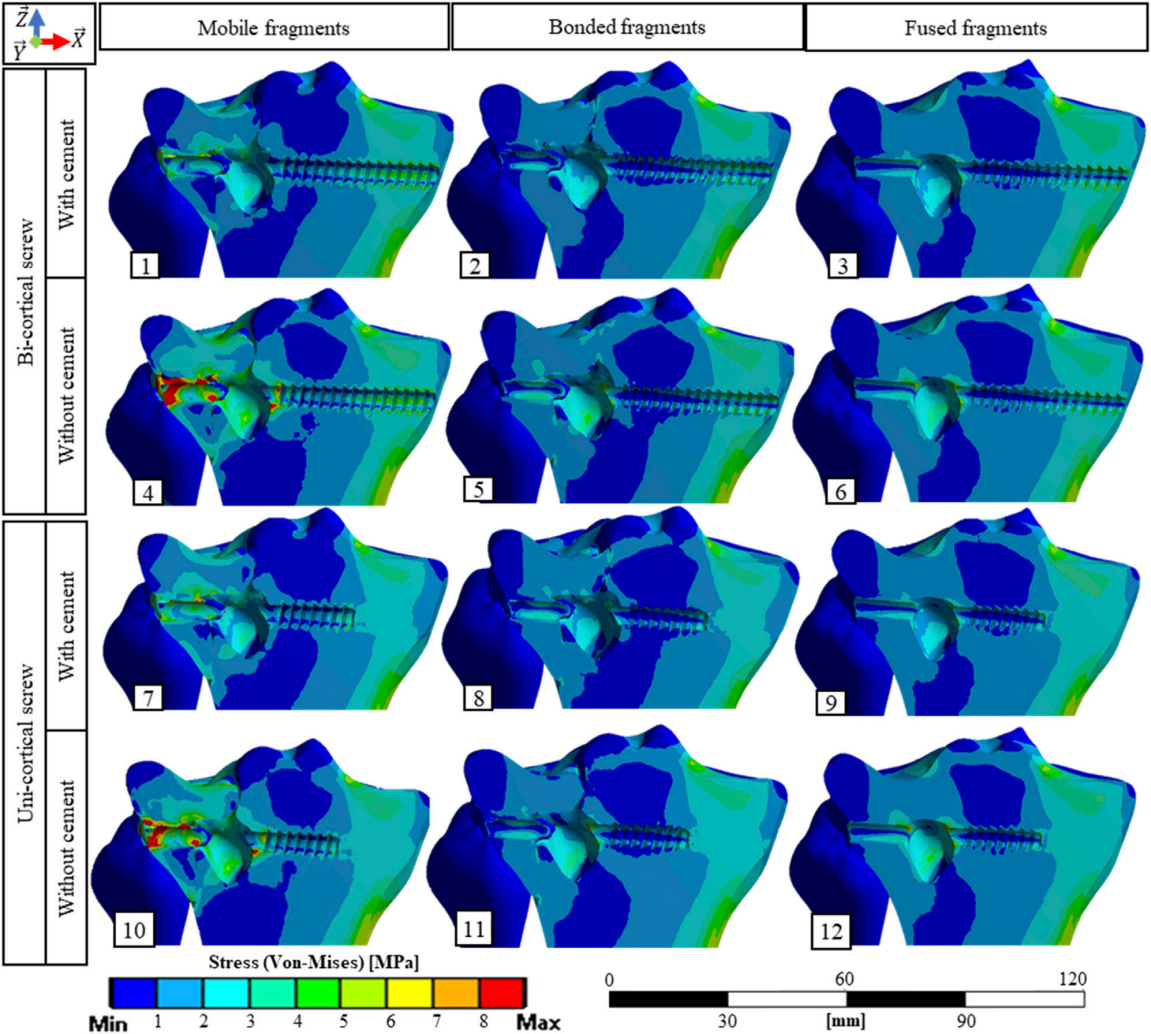
Stress fields (von Mises) in bone material on a cross section containing the screw axis for the 12 simulated scenarios. The maximum stress values in the bone for each scenario are presented Figure 5A.

**FIGURE 4:**
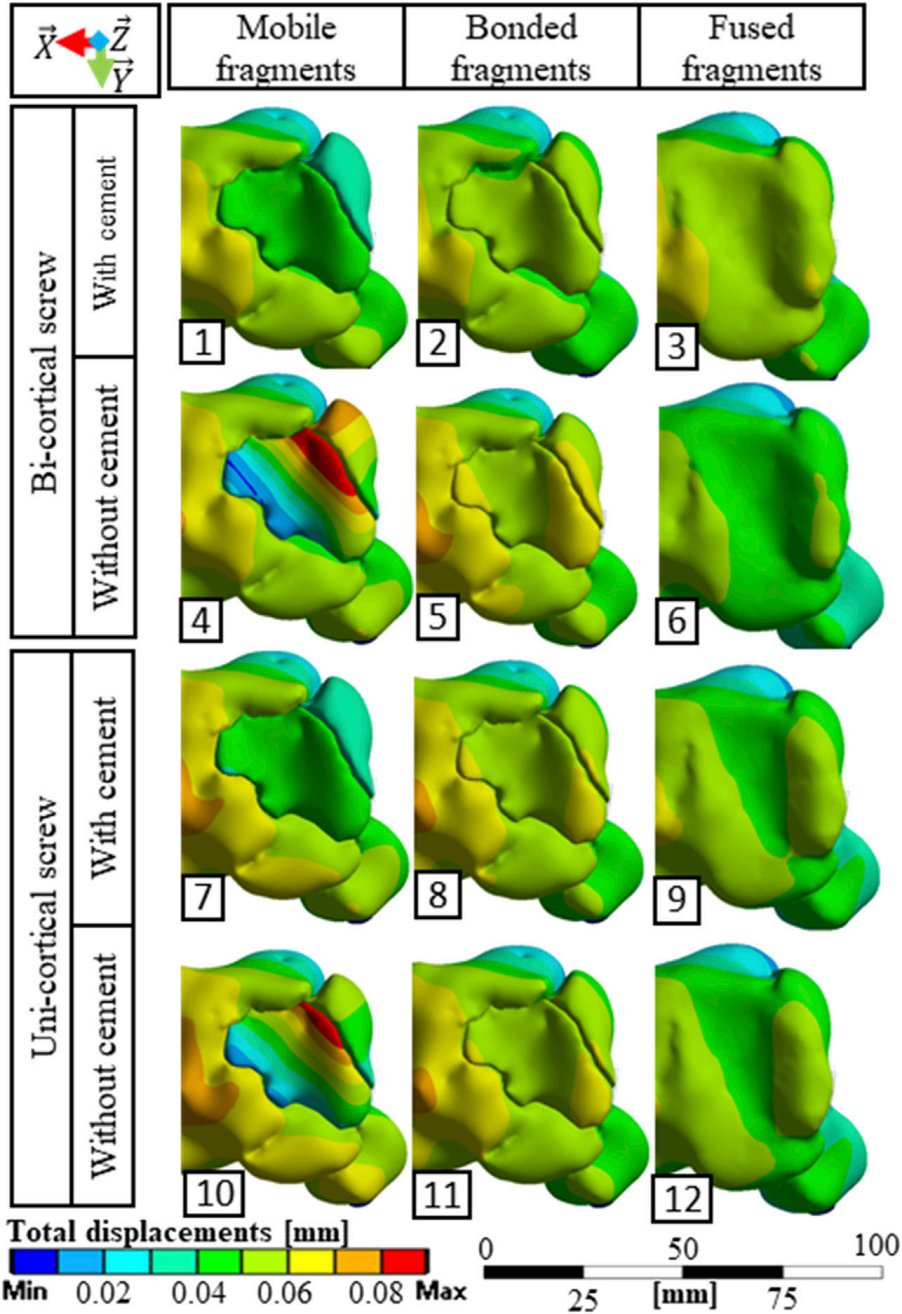
Total displacement fields observed from a superior point of view for the 12 simulated scenarios.

**FIGURE 5:**
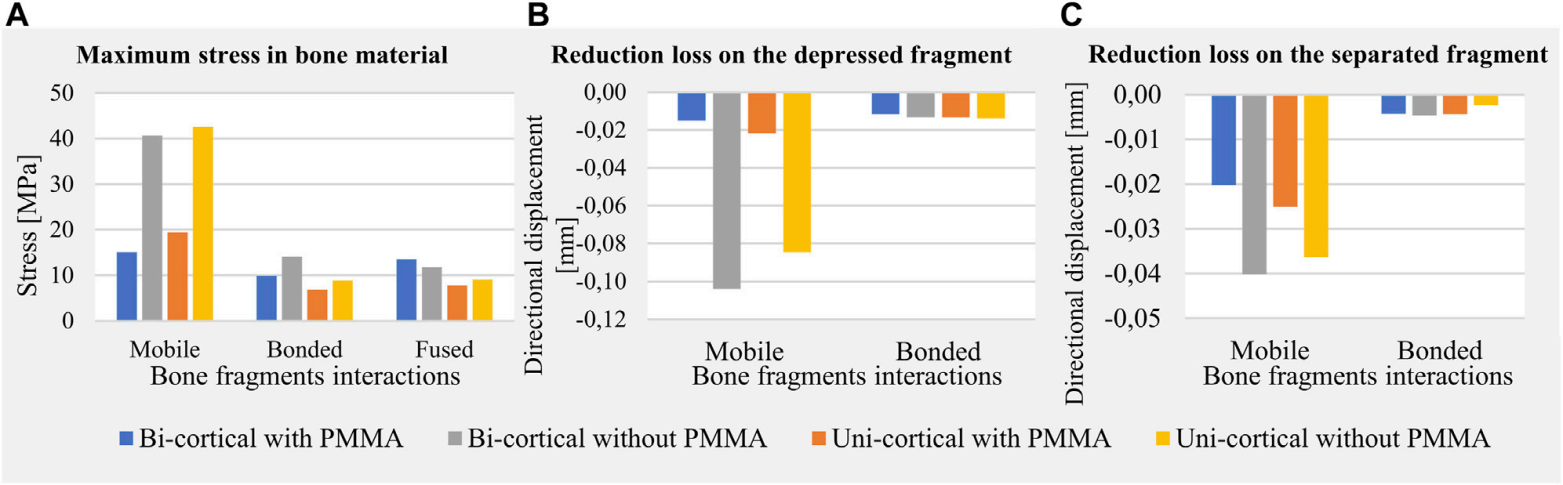
Maximum values of mechanical fields including stress in bone material **(A)**, reduction loss on the depressed fragment expressed as directional displacements **(B)**, and reduction loss on the separated fragment expressed as directional displacements **(C)**.

Stress distributions and displacement fields are, respectively, presented in Figures 3, 4 for the twelve simulated scenarios. In all the scenarios, surgical screws and PMMA cement showed maximum stress below 20% of the corresponding material yield strength. Maximum stresses in bone are presented in Figure 5A. The maximum total displacement was 0.11 mm. It has been observed on the depressed fragment for the simulation with identification number 4 (Figure 4). We measured reduction loss and separate fragment opening the for each scenario (Figures 5B,C). Maximum interfragmentary strains and the volume of bone with stress above the local yield strength are presented in Table 3.

**TABLE 3:** Quantitative assessment of stabilization methods on the tibial plateau fracture stability.

Screw	Cement injection	Fragment interactions	Maximum interfragmentary strains		Bone volume with stress above local yield strength [mm^3^]
			Depressed fragment	Separated fragment	All bone geometries
Bi-cortical Asnis III cannulated screw, Stryker	Yes	Mobile	0.84%	0.58%	0.00
		Bonded	0.65%	0.12%	0.00
		Fused	—	—	0.00
	No	Mobile	5.84%	1.15%	16.18
		Bonded	0.74%	0.13%	0.00
			Fused	—	—	0.00
Uni-cortical Asnis III cannulated screw, Stryker	Yes	Mobile	1.22%	0.72%	0.00
		Bonded	0.74%	0.12%	0.00
		Fused	—	—	0.00
	No	Mobile	3.42%	1.04%	21.66
		Bonded	0.78%	0.07%	0.00
			Fused	—	—	0.00

The stabilization performed by the surgeons included a bi-cortical screw and cement injection. For this scenario, maximum stress experienced in bone was 1.5 times higher with mobile fragments than with bonded fragments; however, no volume of the bone was found with local stress above the local yield strength (Table 3). With this stabilization method, stress distributions were nearly identical with mobile, bonded, and the fused bone fragments (Figure 3). Moreover, the displacement field analysis showed limited reduction loss at all bone healing stages (Figures 5B,C).

Stabilization with uni-cortical screw and cement injection did not show any volume of the bone with stress above local yield strength (Table 3). In this scenario, the maximum stress on the bone was 2.83 times higher with mobile fragments than bonded fragments (Figure 5A).

In the stabilization scenarios without cement injection, we found a volume of the bone with stress above local yield strength of 16.18 and 21.66 mm^3^ with a bi-cortical and a uni-cortical screw, respectively (Table 3). Stress field analysis showed high stresses on the separated and depressed fragments near the screw (Figure 3). These two stabilization methods led to the highest reduction loss when associated with mobile bone fragments (Figure 5B). With bonded and fused bone fragments, no volume of the bone with stress above local yield stress was found (Table 3).

## Discussion

This study presents an original workflow for patient-specific FE modeling and simulation to evaluate variants of stabilization methods at different stages of the bone healing after a tibial plateau fracture. The workflow provides significant and useful models to visualize and quantify the mechanical fields associated with different stabilization methods for and postoperative treatment.

### Stabilization Procedure Performed by the Surgeon

The stabilization procedure performed by the surgeon was composed of one bi-cortical screw and PMMA cement injection. The simulation of this scenario confirms fracture stability for mobile, bonded, and fused bone fragments conditions according to the volume of the bone with stress above the local yield strength, the reduction loss, and interfragmentary strains. Indeed, in their definition of “diamond concept” of bone fracture remodeling, Giannoudis et al. (2007) expressed the importance of absolute or relative fracture stability to ensure bone healing. They reported that interfragmentary strains should be kept below 10%. In our study, all scenarios fulfilled this criterion (Table 3).

### Early Full Weight-Bearing

Full weight-bearing corresponds to the first time the patient can walk again. After surgery of Schatzker type II tibial plateau fracture, the AO Foundation–Orthopedic Trauma Association (AO/OTA) recommends full weight-bearing ten to twelve weeks after the surgery (Ruedi and Murphy, 2000). However, a survey including 111 German orthopedic surgeons showed that 72.1% of them do not follow the AO/OTA recommendations (Van Der Vusse et al., 2017). It is crucial that patients be able to support their weight as soon as feasible, the objective being to maintain bone density, bone healing, and fracture stability (Smet et al., 2005; Giannoudis et al., 2007). In particular, for elderly patients, earlier full weight-bearing is even more decisive due to the health alteration induced by prolonged bed rest complications (Brown et al., 2004). Besides, in a professionally active population, Kraus et al. (2018) showed the negative impact of the tibial plateau fracture on the patient’s subsequent physical ability to work. According to our model, we found two stabilization variants that could allow earlier full weight-bearing without a risk of repeated fractures. They both include PMMA cement injection, associated with a bi-cortical or uni-cortical screw (Table 3).

### PMMA Cement Injection and Screw Length

For younger patients, surgeons must find the best compromise between fracture stability and invasiveness of surgery. The choice of a resorbable cement, presenting low mechanical strength, needs the use of a long construct as described by Hahnhaussen et al. (2012) where a filling with hydroxyapatite cement is proposed with a synthesis plate and eight screws. Since PMMA cement is neither removable nor resorbable and its long-term interactions with the bone remain unclear (Vaishya et al., 2013), determining the influence of PMMA cement injection on fracture stability remains necessary (Belaid et al., 2018). In our patient-specific study, we found that no cement injection increases stresses in bone structures (Figure 5A) and leads to higher risk of repeated fractures in early full weight-bearing scenarios (i.e., mobile bone fragments) (Table 3). Moreover, cement injection remains necessary to decrease reduction loss (Figure 5B). Full weight-bearing at an intermediary bone healing stage (i.e., bonded bone fragments) without cement injection presents no risk of repeated fractures as no volume of the bone with stress above the local yield strength has been found (Table 3). For younger patients for whom priority can be given to reducing invasiveness, stabilization requiring a longer period of convalescence could lead to less invasiveness.

We included screw length as a parameter of the model to characterize its mechanical effect. This was motivated by the preoperative challenge of finding the satisfactory screw length that would guarantee bi-cortical support and limit screw overhang on surrounding soft tissues. Indeed, implantation of a medio-lateral bi-cortical screw in the tibial plateau can conflict with soft tissues, resulting in pain. Dougherty et al. (2008) compared the biomechanical properties of bi-cortical and uni-cortical screws with a locking plate in the cadaveric model of proximal tibial fracture type Schatzker VI. The authors underlined the interest of uni-cortical screws, which enable manufacturers to reduce the number of screw sizes in inventory and allow self-drilling screws to facilitate insertion. In our simulations, the uni-cortical screw slightly increases stress in the bone and PMMA cement compared to the bi-cortical screw, without constituting a risk of repeated fractures. Moreover, the interest of bi-cortical screw compared to uni-cortical screw on the separated fragment motion was negligible (Figure 5C). According to our model, for this patient-specific case of tibial plateau fracture type Schatzker II, bi-cortical anchorage was not necessary insofar as the simulated uni-cortical screw provided equivalent stiffness. The uni-cortical screw appears mechanically sufficient, with a reduced risk of soft tissue lesion and pain.

### Long-Term Scenarios

In long-term scenarios with full bone remodeling modeled by fragments fused and interfragmentary gaps filled, the stress distributions and maximum stresses observed in screw, PMMA, and the bone were equivalent for all stabilization methods (Figure 3). Stress distribution in the bone did not reveal any discontinuities, so the normal bone remodeling according to Wolff’s law could proceed (Wolff, 1986).

### Limitations and Validity

This study presents a personalized digital twin of the tibial plateau fracture with which biomechanical interest of stabilization variants at different stages of bone healing can be quantified. However, this study is not free of limitations. Despite the simulation of twelve scenarios modeling four stabilization methods and three bone healing conditions, only one loading has been simulated. This loading corresponds to the maximum load during walking activity. Other activities involving higher loading as sitting or climbing stairs could be simulated. Moreover, dynamic effects associated with walking activities have not been simulated. In the scenario with mobile fragments, the assumption of frictionless contacts between the bone structures is made. This is motivated by the difficulty to precisely determine a friction coefficient. However, considering that the bone fragments are partially or totally immersed in body fluids, this assumption should be suitable. In future studies, the determination of a friction coefficient should be investigated. Another limitation of our study is the lack of soft tissue. Indeed, due to soft tissue constriction, displacement of the separated and depressed fragment may be lower than the simulated one. In addition, the bone remodeling was simulated with only three stages. A more complex definition including bone healing according to stress distribution could be implemented in the future.

As the presented model is patient-specific, no direct validation of the model with the specific geometries and material properties could be performed. However, the simulated mechanical behaviors with mobile fragments corroborate with previously published numerical and experimental studies on cadaveric specimens (Carrera et al., 2016, 2018; Belaid et al., 2018; Vendeuvre et al., 2018). Further validation including *ex vivo* experimentations, a retrospective and prospective analysis should be conducted before any patient-specific clinical use. A randomized, controlled, and blinded clinical study currently in progress regarding tuberoplasty will provide valuable data to evaluate the presented workflow (Vendeuvre et al., 2019).

## Conclusion

In conclusion, this study presents a patient-specific approach to model stabilization method variants for tibial plateau fracture surgery at different stages of bone healing. It shows the interest of digital twins to provide quantitative and valuable information for clinical decision-making. Rational parameters such as mechanical strength and interfragmentary strains were evaluated to quantify risk of repeated fractures. Moreover, the mechanical fields associated with stabilization variants were also evaluated. The results confirmed the mechanical contribution of cement injection and showed the benefit to use uni-cortical screw, rather than bi-cortical screw. Now developed postoperatively, the workflow we have outlined could provide worthwhile information for preoperative planning tasks. Thanks to digital twins, clinicians could choose the optimal stabilization method depending on the patient characteristics and choose the optimal postoperative treatment. In future studies, the presented workflow should be investigated for other type of fractures.

## Data Availability

The raw data supporting the conclusions of this article will be made available by the authors, without undue reservation.

## References

[B1] Belaid D. Germaneau A. Bouchoucha A. Brémand F. Brèque C. Rigoard P. Vendeuvre T. (2017). Finite Element Analysis of Mechanical Behavior of Stabilization Techniques for Tibial Plateau Fractures. Comput. Methods Biomech. Biomed. Eng. 20, S13–S14. 10.1080/10255842.2017.1382837 29088628

[B2] Belaid D. Vendeuvre T. Bouchoucha A. Brémand F. Brèque C. Rigoard P. Germaneau A. (2018). Utility of Cement Injection to Stabilize Split-Depression Tibial Plateau Fracture by Minimally Invasive Methods: A Finite Element Analysis. Clin. Biomech. 56, 27–35. 10.1016/j.clinbiomech.2018.05.002 29777960

[B3] Brown C. J. Friedkin R. J. Inouye S. K. (2004). Prevalence and Outcomes of Low Mobility in Hospitalized Older Patients. J. Am. Geriatr. Soc. 52, 1263–1270. 10.1111/j.1532-5415.2004.52354.x 15271112

[B4] Buckley R. E. Moran C. G. Apivatthakakul T. (2017). AO Principles of Fracture Management. Vol. 1: Principles, Vol. 2: Specific Fractures. Switzerland: George Thieme Verlag.

[B5] Burkhart T. A. Andrews D. M. Dunning C. E. (2013). Finite Element Modeling Mesh Quality, Energy Balance and Validation Methods: A Review with Recommendations Associated with the Modeling of Bone Tissue. J. of Biomech. 46, 1477–1488. 10.1016/j.jbiomech.2013.03.022 23623312

[B6] Carrera I. Gelber P. E. Chary G. Gomez Masdeu M. González Ballester M. A. Monllau J. C. Noailly J. (2018). An Intact Fibula May Contribute to Allow Early Weight Bearing in Surgically Treated Tibial Plateau Fractures. Knee Surg. Sports Traumatol. Arthrosc. 26, 756–761. 10.1007/s00167-017-4428-7 28255659

[B7] Carrera I. Gelber P. E. Chary G. González-Ballester M. A. Monllau J. C. Noailly J. (2016). Fixation of a Split Fracture of the Lateral Tibial Plateau with a Locking Screw Plate Instead of Cannulated Screws Would Allow Early Weight Bearing: a Computational Exploration. Int. Orthopaedics (Sicot) 40, 2163–2169. 10.1007/s00264-015-3106-y 26780714

[B8] Cook R. B. Curwen C. Tasker T. Zioupos P. (2010). Fracture Toughness and Compressive Properties of Cancellous Bone at the Head of the Femur and Relationships to Non-invasive Skeletal Assessment Measurements. Med. Eng. Phys. 32, 991–997. 10.1016/j.medengphy.2010.06.014 20674457

[B9] De Smet E. Jaecques S. Vandamme K. Vander Sloten J. Naert I. (2005). Positive Effect of Early Loading on Implant Stability in the Bi-cortical guinea-pig Model. Clin. Oral Implants Res. 16, 402–407. 10.1111/j.1600-0501.2005.01156.x 16117763

[B10] Dehoust J. Münch M. Seide K. Barth T. Frosch K.-H. (2020). Biomechanical Aspects of the Posteromedial Split in Bicondylar Tibial Plateau Fractures-A Finite-Element Investigation. Eur. J. Trauma Emerg. Surg. 46, 1257–1266. 10.1007/s00068-020-01538-3 33179130

[B11] Dougherty P. J. Kim D.-G. Meisterling S. Wybo C. Yeni Y. (2008). Biomechanical Comparison of Bicortical Versus Unicortical Screw Placement of Proximal Tibia Locking Plates: A Cadaveric Model. J. Orthop. Trauma 22, 399–403. 10.1097/BOT.0b013e318178417e 18594304

[B12] Elsoe R. Larsen P. Nielsen N. P. H. Swenne J. Rasmussen S. Ostgaard S. E. (2015). Population-based Epidemiology of Tibial Plateau Fractures. Orthopedics 38, e780–e786. 10.3928/01477447-20150902-55 26375535

[B13] Giannoudis P. V. Einhorn T. A. Marsh D. (2007). Fracture Healing: The diamond Concept. Injury 38 Suppl 4, S3–S6. 10.1016/S0020-1383(08)70003-2 18224731

[B14] Hahnhaussen J. Hak D. J. Weckbach S. Heiney J. P. Stahel P. F. (2012). Percutaneous Inflation Osteoplasty for Indirect Reduction of Depressed Tibial Plateau Fractures. Orthopedics 35, 768–772. 10.3928/01477447-20120822-04 22955384

[B15] Havaldar R. Pilli S. Putti B. (2014). Insights into the Effects of Tensile and Compressive Loadings on Human Femur Bone. Adv. Biomed. Res. 3, 101. 10.4103/2277-9175.129375 24800190 PMC4007336

[B16] Hofman M. Koopmans G. Kobbe P. Poeze M. Andruszkow H. Brink P. R. G. Pape H.-C. (2015). Improved Fracture Healing in Patients with Concomitant Traumatic Brain Injury: Proven or Not? Mediators of Inflamm. 2015, 1–14. 10.1155/2015/204842 PMC4385630 25873754

[B17] Huang X. Zhi Z. Yu B. Chen F. (2015). Stress and Stability of Plate-Screw Fixation and Screw Fixation in the Treatment of Schatzker Type IV Medial Tibial Plateau Fracture: a Comparative Finite Element Study. J. Orthop. Surg. Res. 10, 182. 10.1186/s13018-015-0325-2 26608217 PMC4658795

[B18] Ingrassia T. Nigrelli V. Pecorella D. Bragonzoni L. Ricotta V. (2020). Influence of the Screw Positioning on the Stability of Locking Plate for Proximal Tibial Fractures: A Numerical Approach. Appl. Sci. 10, 4941. 10.3390/app10144941

[B19] Kfuri M. Schatzker J. (2018). Revisiting the Schatzker Classification of Tibial Plateau Fractures. Injury 49, 2252–2263. 10.1016/j.injury.2018.11.010 30526924

[B20] Kikinis R. Pieper S. D. Vosburgh K. G. (2014). “3D Slicer: A Platform for Subject-Specific Image Analysis, Visualization, and Clinical Support,” in Intraoperative Imaging And Image-Guided Therapy. Editor Jolesz F. A. (New York, NY: Springer New York), 277–289. 10.1007/978-1-4614-7657-3_19

[B21] Kraus T. M. Abele C. Freude T. Ateschrang A. Stöckle U. Stuby F. M. Schröter S. (2018). Duration of Incapacity of Work after Tibial Plateau Fracture Is Affected by Work Intensity. BMC Musculoskelet. Disord. 19, 281. 10.1186/s12891-018-2209-1 30086739 PMC6081854

[B22] Lacroix D. Prendergast P. J. (2002). A Mechano-Regulation Model for Tissue Differentiation during Fracture Healing: Analysis of gap Size and Loading. J. of Biomech. 35, 1163–1171. 10.1016/S0021-9290(02)00086-6 12163306

[B23] Lee C. (2005). “The Mechanical Properties of PMMA Bone Cement,” in The Well-Cemented Total Hip Arthroplasty: Theory and Practice. Editors Breusch S. Malchau H. (Berlin, Heidelberg: Springer), 60–66. 10.1007/3-540-28924-0_6

[B24] Mehnert A. Jackway P. (1997). An Improved Seeded Region Growing Algorithm. Pattern Recognition Lett. 18, 1065–1071. 10.1016/S0167-8655(97)00131-1

[B25] Meinberg E. Agel J. Roberts C. Karam M. Kellam J. (2018). Fracture and Dislocation Classification Compendium-2018. J. Orthop. Trauma 32, S1–S10. 10.1097/BOT.0000000000001063 29256945

[B26] Nazemi S. M. Amini M. Kontulainen S. A. Milner J. S. Holdsworth D. W. Masri B. A. Wilson D. R. Johnston J. D. (2015). Prediction of Local Proximal Tibial Subchondral Bone Structural Stiffness Using Subject-specific Finite Element Modeling: Effect of Selected Density-Modulus Relationship. Clin. Biomech. 30, 703–712. 10.1016/j.clinbiomech.2015.05.002 26024555

[B27] No Y. J. Roohani-Esfahani S.-I. Zreiqat H. (2014). Nanomaterials: the Next Step in Injectable Bone Cements. Nanomedicine 9, 1745–1764. 10.2217/nnm.14.109 25321173

[B28] Poh S.-Y. Yew K.-S. A. Wong P.-L. K. Koh S.-B. J. Chia S.-L. Fook-Chong S. Howe T.-S. (2012). Role of the Anterior Intermeniscal Ligament in Tibiofemoral Contact Mechanics during Axial Joint Loading. The Knee 19, 135–139. 10.1016/j.knee.2010.12.008 21257313

[B29] Rho J. Y. Hobatho M. C. Ashman R. B. (1995). Relations of Mechanical Properties to Density and CT Numbers in Human Bone. Med. Eng. Phys. 17, 347–355. 10.1016/1350-4533(95)97314-F 7670694

[B30] Rozell J. C. Vemulapalli K. C. Gary J. L. Donegan D. J. (2016). Tibial Plateau Fractures in Elderly Patients. Geriatr. Orthop. Surg. Rehabil. 7, 126–134. 10.1177/2151458516651310 27551570 PMC4976737

[B31] Ruedi T. P. Murphy W. M. (2000). AO Principles of Fracture Management. AO Princ. Fract. Manag., 868.

[B32] Scott C. E. H. Simpson A. H. R. W. Pankaj P. (2020). Distinguishing Fact from Fiction in Finite Element Analysis. Bone Jt. J. 102-B, 1271–1273. 10.1302/0301-620X.102B10.BJJ-2020-0827.R1 32993345

[B33] Snyder S. M. Schneider E. (1991). Estimation of Mechanical Properties of Cortical Bone by Computed Tomography. J. Orthop. Res. 9, 422–431. 10.1002/jor.1100090315 2010847

[B34] Vaishya R. Chauhan M. Vaish A. (2013). Bone Cement. J. of Clin. Orthopaedics Trauma 4, 157–163. 10.1016/j.jcot.2013.11.005 PMC3880950 26403875

[B35] Van Der Vusse M. Kalmet P. H. S. Bastiaenen C. H. G. van Horn Y. Y. Brink P. R. G. Seelen H. A. M. (2017). Is the AO Guideline for Postoperative Treatment of Tibial Plateau Fractures Still Decisive? A Survey Among Orthopaedic Surgeons and Trauma Surgeons in the Netherlands. Arch. Orthop. Trauma Surg. 137, 1071–1075. 10.1007/s00402-017-2718-7 28534233 PMC5511292

[B36] Vendeuvre T. Babusiaux D. Brèque C. Khiami F. Steiger V. Merienne J.-F. Scepi M. Gayet L. E. (2013). Tuberoplasty: Minimally Invasive Osteosynthesis Technique for Tibial Plateau Fractures. Orthopaedics Traumatol. Surg. Res. 99, S267–S272. 10.1016/j.otsr.2013.03.009 23622864

[B37] Vendeuvre T. Grunberg M. Germaneau A. Maloubier F. Faure J.-P. Gayet L.-E. Rigoard P. Brèque C. (2018). Contribution of Minimally Invasive Bone Augmentation to Primary Stabilization of the Osteosynthesis of Schatzker Type II Tibial Plateau Fractures: Balloon vs Bone Tamp. Clin. Biomech. 59, 27–33. 10.1016/j.clinbiomech.2018.08.004 30142475

[B38] Vendeuvre T. Monlezun O. Brandet C. Ingrand P. Durand-Zaleski I. Gayet L.-E. Germaneau A. Khiami F. Roulaud M. Herpe G. Rigoard P. (2019). Comparative Evaluation of Minimally Invasive 'tibial Tuberoplasty' Surgical Technique versus Conventional Open Surgery for Schatzker II-III Tibial Plateau Fractures: Design of a Multicentre, Randomised, Controlled and Blinded Trial (TUBERIMPACT Study). BMJ Open 9, e026962. 10.1136/bmjopen-2018-026962 PMC6731842 31481365

[B39] Wolff J. (1986). “Concept of the Law of Bone Remodelling,” in The Law of Bone Remodelling. Editor Wolff J. (Berlin, Heidelberg: Springer), 1. 10.1007/978-3-642-71031-5_1

[B40] Zhao D. Banks S. A. Mitchell K. H. D'Lima D. D. Colwell C. W. Fregly B. J. (2007). Correlation between the Knee Adduction Torque and Medial Contact Force for a Variety of Gait Patterns. J. Orthop. Res. 25, 789–797. 10.1002/jor.20379 17343285

